# Diagnostic Complexity and Long-Term Management of Gastrointestinal Stromal Tumor Mimicking Ovarian Origin: A Case Report

**DOI:** 10.7759/cureus.64523

**Published:** 2024-07-14

**Authors:** Chahat Singh, Pankaj Gharde, Pravin W Nikhade, Meen M Morey, Bhagyesh Sapkale

**Affiliations:** 1 General Surgery, Jawaharlal Nehru Medical College, Datta Meghe Institute of Higher Education and Research, Wardha, IND; 2 General Surgery, Datta Meghe Medical College, Datta Meghe Institute of Higher Education and Research, Nagpur, IND; 3 Medicine, Jawaharlal Nehru Medical College, Datta Meghe Institute of Higher Education and Research, Wardha, IND

**Keywords:** surgical excision, ovarian cysts, abdominal mass, exploratory laparotomy, postmenopausal female, histopathology, mesenteric component, ovarian malignancy, gist, gastrointestinal stromal tumor

## Abstract

Gastrointestinal stromal tumors (GISTs) are rare mesenchymal tumors occurring in the gastrointestinal tract particularly the stomach or small intestine originating from interstitial cells of Cajal. This case report describes a 50-year-old postmenopausal female presenting with a gradually increasing abdominal mass which clinically was thought to be a neoplasm originating in the ovaries. A clinical and imaging diagnosis of primary ovarian malignancy was made but during laparotomy, a mesenteric component to the malignancy as well as bilateral ovarian cysts were seen. The mass was removed with care and histopathological analysis confirmed it to be GIST. Follow-up of the patient was done for three years and there was no sign of any disease in the patient and she had an uncomplicated postoperative period. This case describes the intricacy of GISTs’ diagnosis, the significance of detailed intraoperative analysis, and appropriate postoperative surveillance. Differences and similarities with other similar cases shed light on how such patients present themselves for treatment, thus encouraging differentiated care. Supervisory care is therefore vital in the monitoring of the patient for prolonged periods and to check for any relapse.

## Introduction

A tumor is defined as a new growth of tissue that is characterized by uncontrolled growth coming from abnormal cells or cells that do not die when they should [1]. Tumors can be either benign (non-cancerous) or malignant (cancerous) [2]. Benign tumors are known to not invade the surrounding tissues or other parts of the body [2]. They are deemed less dangerous because they are encapsulated, cannot invade other tissues of the body, and cannot spread in the body through the blood and lymphatic system [1,3]. Tumors that are believed to be life-threatening are commonly known as cancer.

A gastrointestinal stromal tumor (GIST) is a category of cancer that starts in cells of the digestive system specifically in the stomach or small intestine [4]. GISTs are derived from interstitial cells of Cajal that are located in the wall of the gastrointestinal tract, are part of the enteric division of the autonomic nervous system, and their primary function is to regulate peristalsis, the motor function that propels food through the gastrointestinal tract [1, 5].

## Case presentation

**Figure 1:**
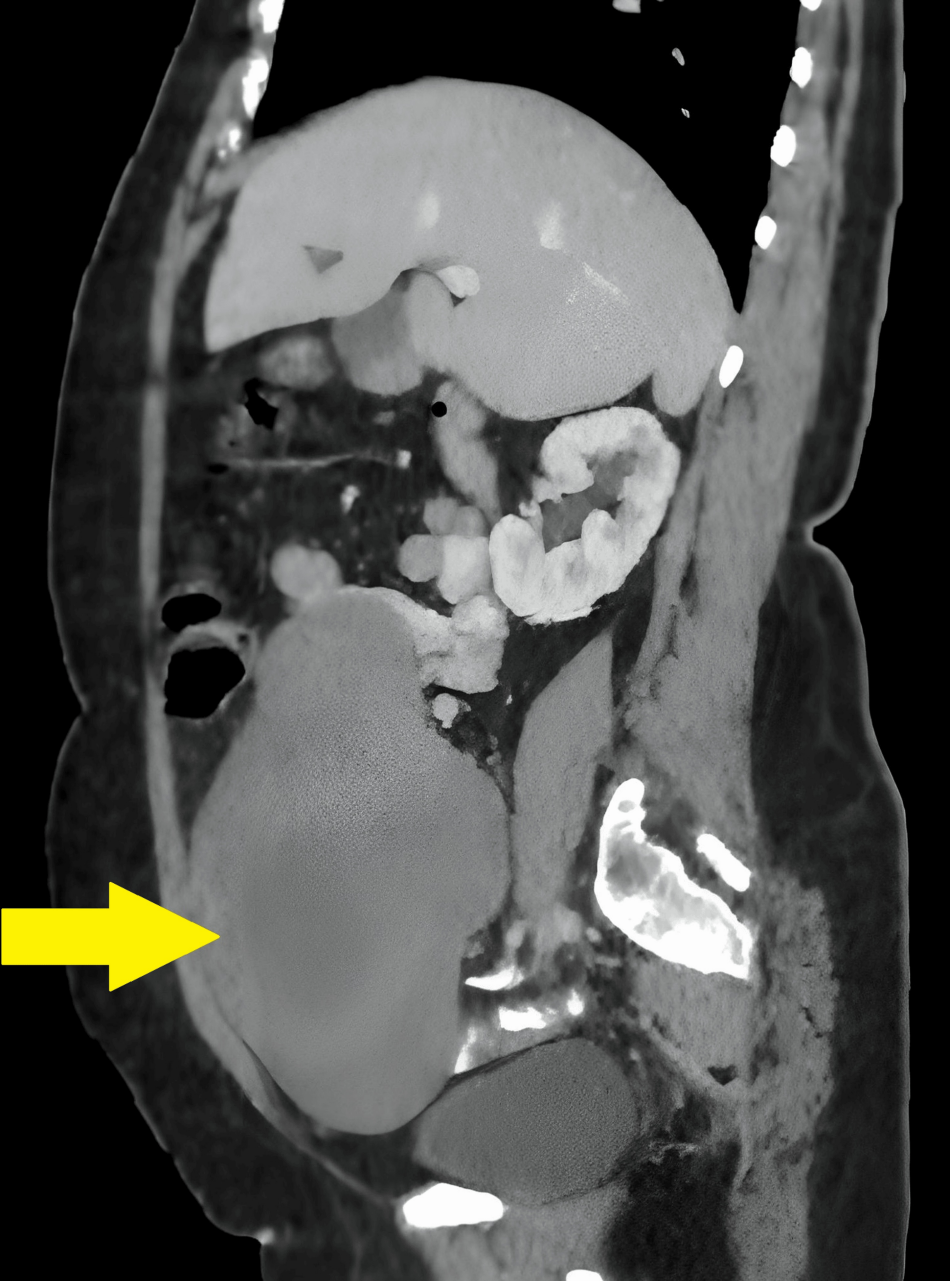
CECT of the abdomen showing the tumor mass (yellow arrow) CECT: contrast-enhanced computed tomography

A 50-year-old postmenopausal female presented with a gradually progressive lump in her abdomen, which had an insidious onset. She reported no associated symptoms such as weight loss or bowel and bladder complaints. Clinical examination and imaging suggested that the mass originated from the pelvis, specifically the ovaries. Contrast-enhanced computed tomography (CECT) scan of the patient is shown in Figure 1.

**Figure 2:**
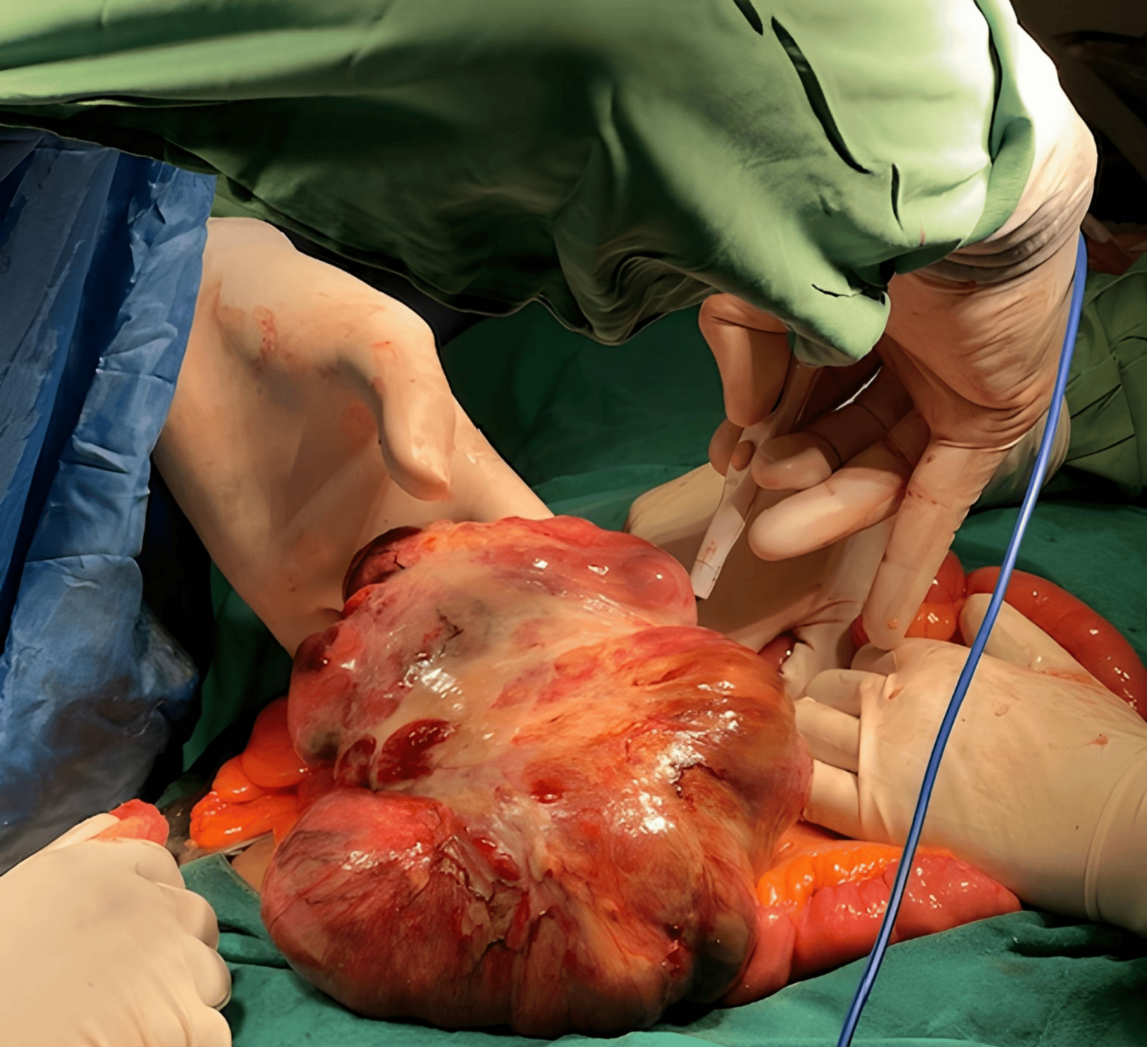
Exploratory laparotomy procedure

Laboratory investigations further supported an ovarian origin. An exploratory laparotomy was performed, which is a surgical procedure where the abdomen is opened and examined to determine the cause of symptoms and the extent of the disease. During this procedure, it was discovered that the abdominal mass had a mesenteric component, meaning it involved the mesentery, the tissue that attaches the intestines to the abdominal wall. Additionally, bilateral ovarian cysts were identified. An image of the exploratory laparotomy procedure is shown in Figure 2.

**Figure 3:**
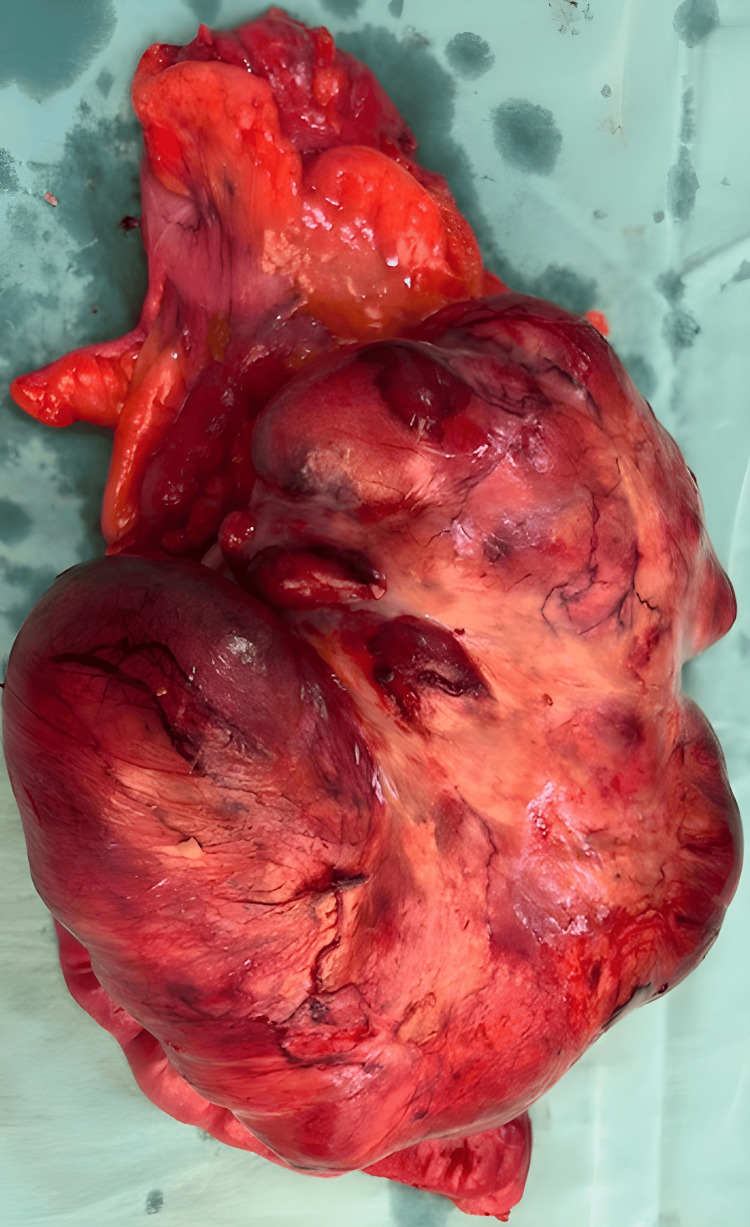
The excised specimen mass

The surgical team proceeded with the excision of the abdominal mass. This involved carefully removing the tumor from the mesentery while preserving as much normal tissue as possible. Given the involvement of the mesentery, the removal of the mass required meticulous dissection to ensure complete resection and to minimize damage to the surrounding structures. The excised specimen mass is shown in Figure 3.

After the extraction of the tumor, an anastomosis was performed. This step was fundamental since removing the tumor required resecting a portion of the intestine, requiring the reconnection of the remaining segments to preserve gastrointestinal continuity and function. Postoperative histopathological examination of the extracted mass affirmed it to be a GIST.

Excised specimen of mesenteric tumor showed bland spindle cells with faintly eosinophilic cytoplasm arranged in a syncytial pattern. Nuclei were round to elongated with inconspicuous nucleoli. Areas of hemorrhage, scanty necrotic material, and proliferating blood vessels were also observed. 

The tumor was present in the mesentery of the intestine, well-circumscribed and partially encapsulated, soft to firm in consistency. The tumor mass, measuring 17.5x17x11 cm, was attached to the ilium, which measured 25 cm. On the cut section, the tumor showed heterogeneous solid, whitish areas, areas of hemorrhage in between, and multiloculated cystic areas filled with 3 serous fluids. The distance of the tumor from the surgical margin was 1.4 cm. Four lymph nodes were identified with the largest measuring 1 x 0.5 cm.

This finding underscored the complexity of the case, as the preoperative evaluations had strongly proposed an ovarian beginning. The patient was closely watched postoperatively for any signs of complications. She had an uneventful recuperation and was discharged from the hospital with normal follow-up courses of action arranged. During such follow-ups for a period of three years, she underwent routine imaging and laboratory examinations and there was no sign of recurrence in the patient, who also remained without any symptoms. She is observed annually to check on her well-being and to be able to identify any late recurrences.

## Discussion

Yamaguchi et al. reported a case of a 53-year-old female who presented with bloating and anorexia and was diagnosed with a massive GIST originating from her stomach and presenting with ovarian and peritoneal metastases [6]. Their patient received a course of imatinib prior to surgery, which significantly reduced the tumor size, and a complex operation, including both open and laparoscopic strategies, was performed to evacuate the tumor. Histopathology affirmed the diagnosis, and the patient remained without any recurrence after 10 months of adjuvant imatinib treatment. The present case, on the other hand, included a 50-year-old postmenopausal female with an abdominal mass that had been dynamically developing. Initial clinical and imaging assessments recommended an ovarian origin but during an exploratory laparotomy, respective ovarian cysts and a mass including the mesentery were found. The tumor was carefully expelled, and histological investigation recognized it as a GIST.

In spite of the fact that both the present case and that of Yamaguchi et al. [6] included middle-aged women with GISTs, the presentations and medications were different. In the latter case [6], the patient's GIST started within the stomach and metastasized to the ovary whereas in the present case, the patient's GIST involved the mesentery with an initial doubt of an ovarian origin. Treatment approaches varied too. Unlike Yamaguchi et al.'s case [6], there were no measures to reduce the size of the tumor before surgery in the present case and the patient recovered completely with no recurrence for three years.

A case detailed by Cheng et al. included a 78-year-old woman with determined vaginal bleeding and difficulty in urinating [7]. Upon examination and imaging, a hard, unmovable mass was found projecting from the vaginal wall, expanding towards the rectum. Initial biopsies were uncertain but further surgical intervention uncovered an 8.5×6×3 cm necrotic tumor, identified minutely as a GIST with high malignancy risk. Postoperative investigation showed strong positivity for CD117, DOG1, and h-Caldesmon, leading to the diagnosis of rectal-origin GIST. The patient experienced imatinib treatment and has remained disease-free for over three years. In comparison, in the present case, a 50-year-old postmenopausal woman presented with a progressive abdominal lump, initially suspected to be of ovarian origin. Clinical and imaging assessments pointed to the pelvis and ovaries, but exploratory laparotomy revealed the mass involved the mesentery, along with bilateral ovarian cysts. Despite the initial ovarian suspicion, the final diagnosis was similar to the case reported by Cheng et al. [7], underscoring the complexity of preoperative evaluations. The patient had an uneventful recovery and has shown no signs of recurrence over three years, with regular follow-up monitoring.

Wang et al. reported the case of a 74-year-old female who presented with worsening abdominal pain over two days, which continued after three months of irregular upper abdominal pain [8]. Physical examination uncovered a non-mobile, ineffectively characterized mass around 10 cm in diameter within the upper left abdomen, with tenderness but no rebound tenderness. Laboratory tests were unremarkable, and imaging studies, including abdominal ultrasonography and CT, recognized a large cystic mass compressing adjoining organs. Exploratory laparotomy uncovered a GIST related to the stomach and spleen, requiring resection of the tumor, proximal stomach, and spleen. Postoperative pathology affirmed a high-grade spindle cell variation GIST with a mitotic index of 10/50 high-power field (HPF) and positive immunohistochemical markers. The patient recovered uneventfully and remained disease-free for 20 months post-surgery. This is similar to our case, in which a 50-year-old postmenopausal female presented with a continuously broadening stomach lump without related systemic indications. Imaging and clinical examination proposed an ovarian origin, which was afterward uncovered to include the mesentery amid exploratory laparotomy. The mass and bilateral ovarian cysts were extracted, and postoperative histopathology affirmed the mass as a GIST. The surgery included meticulous dissection and anastomosis to protect gastrointestinal coherence. During a three-year follow-up period, the patient remains asymptomatic without any complications.

These cases emphasize the significance of cautious intraoperative assessment and postoperative surveillance, as well as the symptomatic troubles and surgical complications related to GISTs.

## Conclusions

The present case report shows challenges that may be faced in the diagnosis of GISTs. The patient described was a 50-year-old female who was referred to the hospital with an abdominal mass with clinical-radiological features of ovarian malignancy. Exploratory laparotomy revealed the presence of a mesenteric component along with bilateral ovarian cysts. The mass was excised and, on histopathology, was confirmed to be a GIST.

This case underlines the fact that despite preoperative assessments, the clinician can be caught off guard during surgery. It also emphasizes the need to properly manage the condition postoperatively in the long term for the patient’s well-being as well as to detect the first signs of the disease if it recurs. In the three years of follow-up, the patient had no sign of the disease.

## References

[1] Schaefer IM, Mariño-Enríquez A, Fletcher JA (2017). What is new in gastrointestinal stromal tumor?. Adv Anat Pathol.

[2] Velilla Vico D, Carbonell Morote S, Ruiz de la Cuesta Tapia E, Ramia Ángel JM (2023). Gastric gastrointestinal stromal tumor abscess. Rev Esp Enferm Dig.

[3] Tan Z (2019). Recent advances in the surgical treatment of advanced gastric cancer: a review. Med Sci Monit.

[4] Sharma AK, Kim TS, Bauer S, Sicklick JK (2022). Gastrointestinal stromal tumor: new insights for a multimodal approach. Surg Oncol Clin N Am.

[5] Stamatakos M, Douzinas E, Stefanaki C, Safioleas P, Polyzou E, Levidou G, Safioleas M (2009). Gastrointestinal stromal tumor. World J Surg Oncol.

[6] Yamaguchi T, Kinoshita J, Saito H (2021). Gastrointestinal stromal tumor metastasis to the ovary: a case report. SAGE Open Med Case Rep.

[7] Cheng M, Liu CH, Horng HC, Chen YJ, Lo PF, Lee WL, Wang PH (2019). Gastrointestinal stromal tumor presenting as a rectovaginal septal mass: a case report and review of literature. Medicine (Baltimore).

[8] Wang L, Liu L, Liu Z, Tian Y, Lin Z (2017). Giant gastrointestinal stromal tumor with predominantly cystic changes: a case report and literature review. World J Surg Oncol.

